# Divergence in Gut Bacterial Community Structure between Male and Female Stag Beetles *Odontolabis fallaciosa* (Coleoptera, Lucanidae)

**DOI:** 10.3390/ani10122352

**Published:** 2020-12-09

**Authors:** Xia Wan, Yu Jiang, Yuyan Cao, Binghua Sun, Xingjia Xiang

**Affiliations:** Anhui Province Key Laboratory of Wetland Ecosystem Protection and Restoration, School of Resources and Environmental Engineering, Anhui University, Hefei 230601, China; wanxia@ahu.edu.cn (X.W.); jiangyu626@163.com (Y.J.); c15155149311@126.com (Y.C.); binghuasun00@126.com (B.S.)

**Keywords:** bacterial community structure, Firmicutes, beetle, sex dimorphism, male trimorphism

## Abstract

**Simple Summary:**

Intestinal microbiota play crucial roles for their hosts. *Odontolabis fallaciosa* shows striking sexual dimorphism and male trimorphism, which represents an interesting system to study their gut microbiota. We have compared the intestinal bacterial community structure between the two sexes and among three male morphs of *O. fallaciosa*. The gut bacterial community structure was significantly different between males and females. The females were associated with higher bacterial alpha-diversity relative to males. Large males had a higher relative abundance of Firmicutes and Firmicutes/Bacteroides (F/B) ratio, which contributed to nutritional efficiency. The results increased our understanding of beetle–bacterial interactions of *O. fallaciosa* between the two sexes, and among three male morphs, which might reveal the relationship among the gut microbiota, nutrition level, and phenotypic evolution of the stag beetle.

**Abstract:**

*Odontolabis fallaciosa* (Coleoptera: Lucanidae) is a giant and popular stag beetle with striking sexual dimorphism and male trimorphism. However, little is known about their intestinal microbiota, which might play an indispensable role in shaping the health of their hosts. The aim of this study was to investigate the intestinal bacterial community structure between the two sexes and among three male morphs of *O. fallaciosa* from China using high-throughput sequencing (Illumina MiSeq). The gut bacterial community structure was significantly different between males and females, suggesting that sex appeared to be the crucial factor shaping the intestinal bacterial community. Females had higher bacterial alpha-diversity than males. There was little difference in gut bacterial community structure among the three male morphs. However, compared to medium and small males, large individuals were associated with the higher relative abundance of Firmicutes and Firmicutes/Bacteroides (F/B) ratio, which might contribute to nutritional efficiency. Overall, these results might help to further our understanding of beetle–bacterial interactions of *O. fallaciosa* between the two sexes, and among the three male morphs.

## 1. Introduction

The intestinal microbiota of animals are composed of densely populated microbial assemblages [1]. The gut microbiota contributes to many necessary host functions, including increasing nutritional efficiency [2], improving host health [3], training the immune system [4], and regulating host physiology [5]. The interaction between hosts and their gut microbiota significantly affects host behaviors [6,7]. Gut microbiota influenced the mate choice of *Drosophila melanogaster* [8] and caused hybrid inviability in *Nasonia* [9]. Gut microbial community patterns are affected by a series of complex and dynamic interactions throughout life, including diet [10], age [11], gender [12], seasonal fluctuations [13], and genotype [14].

Empirical studies have intensively clarified intestinal microbial assemblages in vertebrates [1,15]. Insects are the most abundant species group in the animal kingdom and have unique life history traits. Thus, they represent an interesting system for intestinal microbiota. Intestinal microbiota have been studied in mosquitoes, bees, termites, and cockroaches [16,17,18,19,20] and have shown that gut microbial communities benefited their hosts [21]. Intestinal microbiota help insects digest recalcitrant materials [22], prevent the invasion of parasites and pathogens [23], and aid in intraspecific communication [6,24].

Stag beetles (Coleoptera: Lucanidae) are striking insects due to their large size and prominent mandibles. About 1800 species and subspecies have been described worldwide. Most species inhabit tropical and sub-tropical forests. The larvae live in and feed on decaying wood, while adults utilize tree sap and overripe fruits [25]. The larvae of beetles rely on dead wood for growth, which facilitates wood decomposition, nutrient cycling, and vegetative growth. Thus, they are good biological indicators of forest matter cycling [26,27]. Their gut microbiota are important for converting a food source with low nutritional value into a nutritionally adequate substance. A fungus-storage organ (i.e., mycangium) was found in female *Platycerus* and *Sinodendron* stag beetles [28,29]. Xylose-fermenting yeast in *Scheffersomyces* has been found in the mycangium to help host increase their nutrition levels. In addition, mycangial yeasts can be transmitted vertically from adult females to their larvae [28].

Despite studies showing gut yeast in lucanid species, knowledge about the gut bacterial community structure of beetle species is limited. Moreover, the gut bacterial communities between conspecific adult males and females have not been definitely verified. The yellow-spot stag beetle, *O. fallaciosa*, is saproxylic species distributed in tropical and subtropical forests from northern Vietnam to southern China. Adults of this species exhibit dramatically sexual dimorphism. In addition, the adult males show rare phenotypic trimorphism [30,31]. Three types of male morphs, that is large-, medium-, and small-sized males, coexist and display large variations in mandible shape and body size. Thus, this species offers an excellent opportunity to examine the gut bacterial community structure between the two sexes and among the three male morphs.

There is a strong correlation between the obtained nutrients and the male mandible shape/body size [32]. Mandible shape is highly associated with reproductive success in adult males [33,34,35]. This suggests that efficient conversion of indigestible food into adequate nutrition, with the help of gut microbiota, is essential for the growth of large males. However, we found little difference in gut bacterial diversity among three male morphs of *O. fallaciosa* in our previous study [36]. We expected that certain bacterial taxa which were associated with increasing hosts’ nutritional efficiency would show significant differences among the three male morphs. In this study, we investigated the gut bacterial community structure of male (including three male morphs) and female *O. fallaciosa* by high-throughput sequencing. In particular, we focused on two questions: (i) Are there sex-related differences in the gut bacterial community structure of *O. fallaciosa*? and (ii) Do the certain intestinal bacterial taxa that are associated with nutrition provision show differences among the three male morphs?

## 2. Materials and Methods

### 2.1. Site Selection and Sample Collection

This study was based on 46 adult individuals of *O. fallaciosa*, consisting of 15 females and 31 males. The males included three morphs (i.e., trimorphism) based on mandible shape and body size: large-sized males (LM; 15 individuals), medium-sized males (MM; 10 individuals), and small-sized males (SM; 6 individuals; Figure S1). The beetles were collected between 20–25 July 2016 at Laoshan Mountain, Jinxiu, Guangxi Autonomous Region, P. R. China (24°17′37″ N, 110°25′33″ E). Collection was permitted by the Dept. of Forestry of Guangxi Autonomous Region. Each adult was dissected to collect gut tissues. Prior to collection of gut tissues, the beetles were anesthetized by holding them at −20 °C for 8 min, followed by surface sterilization with 75% alcohol. After the removal of the legs and elytra, the guts were collected by dorsal dissection. All of the gut tissues were stored at −80 °C until processing.

### 2.2. DNA Extraction

DNA extractions were carried out on the guts of *O. fallacoisa* using the Qiagen QIAamp^®^ DNA Stool Mini Kit (Qiagen Inc. Valencia, CA, USA), based on the protocol.

### 2.3. High-Throughput Sequencing

An aliquot (50 ng) of purified DNA was used as a template with primer (F515/R907) to amplify the V4–V5 hypervariable regions of the bacterial 16S rRNA genes fragments for the Illumina MiSeq platform (PE 300) at Majorbio (Shanghai, China) [37]. The detailed parameters for amplicon library preparation are shown in our previous study [1]. The last 50 bp of raw paired-end reads was cut off and then merged using FLASH v.1.2.11 [38]. Quantitative Insights Into Microbial Ecology (QIIME v.1.9.0) was used for further analysis. Sequences below an average quality score of 30 (i.e., low quality) were filtered out. The remaining sequences were clustered into operational taxonomic units (OTUs) at 97% similarity with the usearch algorithm. Chimera check was performed. All of the singleton OTUs were deleted. The most abundant sequence within each OTU was selected as the representative sequence, identified using uclust with Silva v.132 as reference database and aligned with PyNAST [39]. To equally rarefy samples, randomly selected subsets of 8900 sequences per sample were used to compare the bacterial community structure for all samples.

### 2.4. Statistical Analysis

The dominant intestinal bacterial phyla were determined by the mean relative abundance of all individuals > 1% in this study. A one-way analysis of variance (ANOVA) was used to analyze bacterial alpha-diversity and the relative abundance of dominant phyla. The multivariate variances in bacterial community composition were evaluated by betadisper analysis using the vegan package in R 3.4.3 software. The differences in bacterial community compositions were analyzed by non-metric multidimensional scaling (NMDS) and analysis of similarity (ANOSIM) among treatments using the vegan package in R 3.4.3 software. The contribution of genera to the differences of bacterial community composition between male and female beetles was analyzed by SIMPER analysis using the vegan package in R software (v.3.4.3). Indicator analysis was done to identify bacterial OTUs that are specifically associated with the certain treatment using the labdsv package in R software (v.3.4.3).

### 2.5. Data Availability

The raw data were submitted to the Sequence Read Archive (SRA) of National Center for Biotechnology Information (NCBI) under the accession number SRP238193.

## 3. Results

### 3.1. Intestinal Bacterial Alpha-Diversity

**Figure 1:**
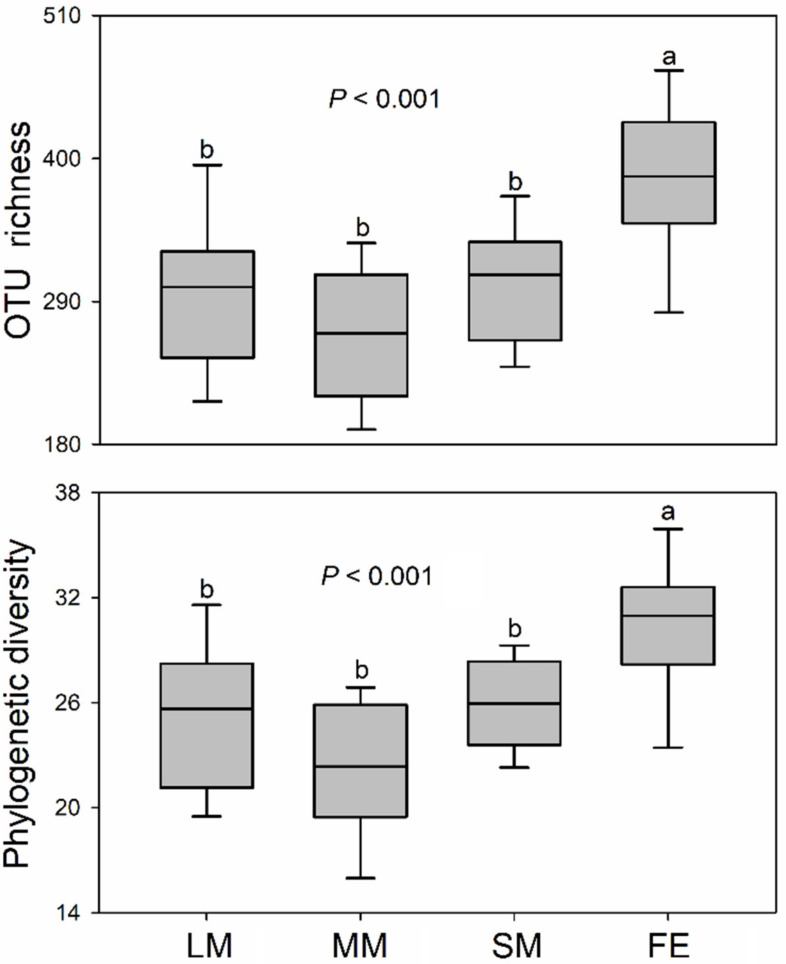
Intestinal bacterial alpha-diversity of *Odontolabis fallaciosa* across different treatments. The bottom and top of the box denote the first and third quartiles; the band inside the box denotes the median; letters above bars represent significant differences from the one-way ANOVA with Tukey’s honestly significant difference (HSD) post-hoc testing (*p* < 0.05). LM: large male; MM: medium-sized male; SM: small male; FE: female; OTU: operational taxonomic unit.

A total of 2552 bacterial OTUs (97% similarity) were found, ranging from 189 to 503 across all samples, 18.3% of which (468) were found in all treatments (Figure S2). The unique bacterial OTUs of *O. fallaciosa* were 450 (17.6%), 264 (10.3%), 130 (5.1%), and 470 (18.4%) for treatments of large-sized males (LM), medium-sized males (MM), small-sized males (SM), and females (FE), respectively (Figure S2). The one-way ANOVA showed that the bacterial alpha-diversity of *O. fallaciosa* (i.e., OTU richness and phylogenetic diversity) was significantly different between males and females (*p* < 0.001), with higher alpha-diversity in females (Figure 1). The intestinal bacterial alpha-diversity did not show any difference among three male morphs (Figure 1).

### 3.2. Intestinal Bacterial Community Structure

**Figure 2:**
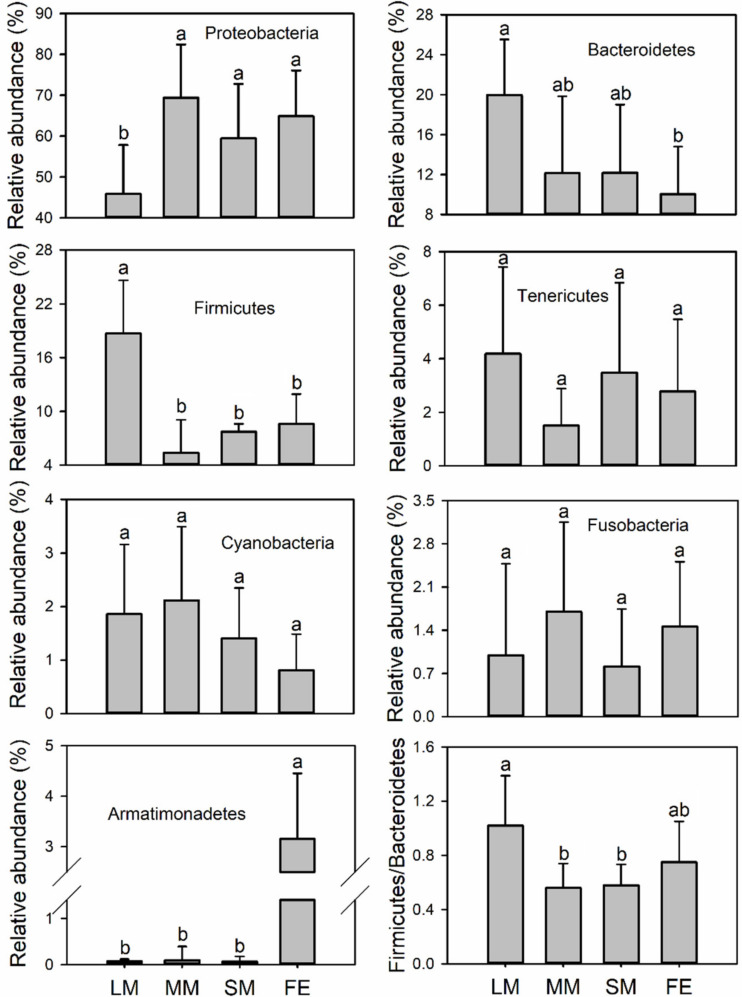
Relative abundances of dominant bacterial phyla of *Odontolabis fallaciosa* across treatments. Error bars denote standard deviation; different letters represent significant differences from a one-way ANOVA with Tukey’s honestly significant difference (HSD) post-hoc testing (*p* < 0.05). LM: large male; MM: medium-sized male; SM: small male; FE: female.

The dominant intestinal bacterial phyla of *O. fallaciosa* were Proteobacteria (58.9%), Bacteroidetes (14.5%), Firmicutes (11.1%), Tenericutes (3.05%), Cyanobacteria (1.51%), Fusobacteria (1.28%), and Armatimonadetes (1.06%) (Figure 2). LMs had a higher relative abundance of Firmicutes and lower relative abundance of Proteobacteria relative to other treatments (Figure 2). The female beetles had a higher relative abundance of Armatimonadetes relative to the males (Figure 2). The ratio of intestinal Firmicutes/Bacteroidetes (F/B) was significantly higher in LMs relative to MMs and SMs (Figure 2). Indicator analysis was used to identify bacterial OTUs that are specifically associated with different treatments. The results showed that there were 10, 1, 3, and 16 indicator species in the LMs, MMs, SM, and FEs, respectively (Table S1).

**Figure 3:**
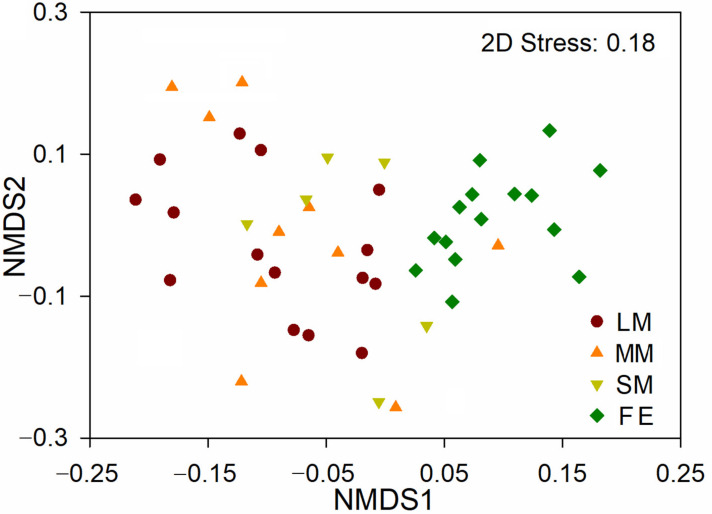
The intestinal bacterial community compositions across different treatments. LM: large male; MM: medium-sized male; SM: small male; FE: female.

Similar multivariate variance was found in the gut bacterial community compositions among different treatments (*p* < 0.05 in all cases; Table S2). The gut bacterial community compositions were significantly different between males and females of *O. fallaciosa* (Table S2; Figure 3). Compared to the females, the gut bacterial community composition of large males (i.e., r = 0.420) showed a greater difference than medium (i.e., r = 0.234) and small (r = 0.267) males. However, there was little difference in the intestinal bacterial community composition among the three male morphs (Table S2; Figure 3). SIMPER analysis was performed to clarify which gut bacterial OTUs primarily contributed to community dissimilarities between the males and females. The results revealed that OTU_782 (*Acinetobacter*; 15.42%), OTU_3378 (*Pseudomonas*; 9.48%), and OTU_117 (*Chryseobacterium*; 6.64%) made primary contributions to the differences in gut bacterial community between the male and female beetles (Table 1).

## 4. Discussion

Dramatic shifts in intestinal bacterial community structures were found between male and female *O. fallaciosa* (Figure 1, Figure 2 and Figure 3), suggesting that sexual variation was a crucial factor shaping the intestinal bacterial structure [40]. The gut bacterial genera *Acinetobacter* (15.42%) and *Pseudomonas* (9.48%) primarily contributed to the differences in bacterial community composition between males and females (Table 1). Previous studies have found that both *Acinetobacter* and *Pseudomonas* helped hosts’ digestion and increased the availability of nutrients [41,42]. However, males showed higher relative abundance of *Acinetobacter*, while females had greater relative abundance of *Pseudomonas*. The result indicated that sexual variations might be closely linked to their gut bacterial functions.

Consistent with previous studies, female beetles had higher gut bacterial diversity than males [40,43,44]. Heritable taxa have been demonstrated in stag beetles [6,45,46], suggesting that higher intestinal bacterial diversity in females might contribute to the vertical transmission (i.e., larva acquire diverse bacterial taxa). A sex-specific metabolic pathway and immune system might be important reasons for differences in bacterial diversity between the two sexes of beetles [40,47,48]. Differences in gut bacterial diversity might also be induced by differences in the preferential diet of beetles between the sexes. However, we did not investigate diet data of the beetles. Thus, the limitation should be clearly clarified in a future study.

Interestingly, there was little difference in gut bacterial community composition and diversity among the three male morphs of *O. fallaciosa* (Figure 1 and Figure 3), suggesting that mandible shape and body size had little effect on their gut bacterial community structure. However, large males were associated with a lower relative abundance of Proteobacteria. Previous studies have demonstrated that many animal pathogens belong to Proteobacteria [49], suggesting that large males might be at lower risk of diseases relative to other male morphs. In addition, we found a higher relative abundance of Firmicutes and Firmicutes/Bacteroidetes (F/B) ratio in large males relative to other males. Intestinal Firmicutes contribute to the decomposition of complex carbohydrates, fatty acids, and polysaccharides [50,51], which might improve the ability of the host to derive nutrients from a nutrient-poor diet [52]. Nutrition level has been proven as the crucial factor to regulate size of body and mandible in male beetles [32,53]. The higher F/B ratio might be of significant relevance to body size by providing a stronger capacity for energy harvest and nutrition provision [54,55,56]. The large male was associated with a higher reproductive success among the three male morphs [35]. Thus, these results suggested that large males were associated with a higher relative abundance of favorable bacterial taxa (i.e., Firmicutes and F/B ratio) and a lower relative abundance of pathogenic taxa (i.e., Proteobacteria), which allow them to increase their nutrition levels and decrease the risk of diseases, resulting in their being more attractive to female beetles.

## 5. Conclusions

Gut bacterial community structure showed significant differences between males and females of *O. fallaciosa*, suggesting that the gut bacterial community of beetles may have strong sexual preferences. The males were associated with higher bacterial diversity, which might contribute to vertical transmission. The bacterial community structure showed little difference among the three male morphs. However, the guts of large males might contain a higher relative abundance of favorable bacterial taxa and lower relative abundance of pathogenic taxa that allows them to increase their nutrition levels and decrease the risk of diseases. This study provided a better picture of beetle–bacterial interactions between the two sexes, and among three male morphs. Future work should focus on the intestinal microbial abundance and community structure at different growth stages to clarify intestinal microbial functions in controlling the life history of stag beetles.

## Figures and Tables

**Table 1:** SIMPER analysis showing the contribution of intestinal bacterial OTUs to the difference between male and female beetles. Taxonomic leaves: o, order; c, class; f, family; g, genus. MA: Male beetle; FE: Female beetle.

OTU	Taxa	Contribution (%)
		MA vs. FE
782	g__*Acinetobacter*	15.42
3378	g__*Pseudomonas*	9.48
117	g__*Chryseobacterium*	6.64
906	f__Moraxellaceae	4.74
2845	f__Enterobacteriaceae	3.99
1435	c__Chthonomonadetes	3.18
434	f__Bartonellaceae	2.56
1184	o__Entomoplasmatales	2.11

## Supplementary Materials

The following are available online at https://www.mdpi.com/2076-2615/10/12/2352/s1, Table S1: Indicator bacterial species in guts of *Odontolabis fallaciosa* across different treatments. Taxonomic leaves: c, class; f, family; g, genus; s, species. LM: large-sized male beetle; MM: medium-sized male beetle; SM: small-sized male beetle; FE: female beetle; Table S2: The multivariate variance across the different treatments was evaluated by betadisper analysis. Differences in gut bacterial community composition across the different treatments examined by the dissimilarity test of ANOSIM. LM: large-sized male beetle; MM: medium-sized male beetle; SM: small-sized male beetle; FE: female beetle. Figure S1: The mandible shape and body size of *O. fallaciosa* between two sexes, and among three male morphs. LM: large-sized male beetle; MM: medium-sized male beetle; SM: small-sized male beetle; FE: female beetle, Figure S2: Venn diagram showing the co-occurrence of OTUs among samples from different treatments. Numbers inside the Venn diagram indicate unique and shared OTUs. OTU, operational taxonomic unit; LM: large-sized male beetle; MM: medium-sized male beetle; SM: small-sized male beetle; FE: female beetle.

## References

[1] Xiang X., Zhang F., Fu R., Yan S., Zhou L. (2019). Significant Differences in Bacterial and Potentially Pathogenic Communities Between Sympatric Hooded Crane and Greater White–Fronted Goose. Front. Microbiol..

[2] Sabree Z.L., Huang C.Y., Arakawa G., Tokuda G., Lo N., Watanabe H., Moran N.A. (2012). Genome shrinkage and loss of nutrient–providing potential in the obligate symbiont of the primitive termite *Mastotermes darwiniensis*. Appl. Environ. Microb..

[3] Hurst G.D.D., Hutchence K.J. (2010). Host defence: Getting by with a little help from our friends. Curr. Biol..

[4] Atarashi K., Tanoue T., Shima T., Imaoka A., Kuwahara T., Momose Y., Cheng G., Yamasaki S., Saito T., Ohba Y. (2011). Induction of Colonic Regulatory T Cells by Indigenous *Clostridium* Species. Science.

[5] Meinl W., Sczesny S., Brigelius–Flohe R., Blaut M., Glatt H. (2009). Impact of Gut Microbiota on Intestinal and Hepatic Levels of Phase 2 Xenobiotic–Metabolizing Enzymes in the Rat. Drug Metab. Dispos..

[6] Engel P., Moran N.A. (2013). The gut microbiota of insects: Diversity in structure and function. FEMS Microbiol. Rev..

[7] Hu Y., Wu Q., Ma S., Ma T., Shan L., Wang X., Nie Y., Ning Z., Yan L., Xiu Y. (2017). Comparative genomics reveals convergent evolution between the bamboo–eating giant and red pandas. Proc. Natl. Acad. Sci. USA.

[8] Sharon G., Segal D., Ringo J.M., Hefetz A., Zilber–Rosenberg I., Rosenberg E. (2010). Commensal bacteria play a role in mating preference of *Drosophila melanogaster*. Proc. Natl. Acad. Sci. USA.

[9] Brucker R.M., Bordenstein S.R. (2013). The hologenomic basis of speciation: Gut bacteria cause hybrid lethality in the genus *Nasonia*. Science.

[10] Bolnick D.I., Snowberg L.K., Hirsch P.E., Lauber C.L., Org E., Parks B., Lusis A.J., Knight R., Caporaso J.G., Svanbäck R. (2014). Individual diet has sex–dependent effects on vertebrate gut microbiota. Nat. Commun..

[11] Mariat D., Firmesse O., Levenez F., Guimarăes V.D., Sokol H., Corthier G., Furet J.-P. (2009). The Firmicutes/Bacteroidetes ratio of the human microbiota changes with age. BMC Microbiol..

[12] Sun B.H., Gu Z.Y., Wang X., Huffman M.A., Garber P.A., Sheeran L.K., Zhang D., Zhu Y., Xia D.P., Li J.H. (2018). Season, age, and sex affect the fecal mycobiota of free–ranging Tibetan macaques (*Macaca thibetana*). Am. J. Primatol..

[13] Dong Y., Xiang X., Zhao G., Song Y., Zhou L. (2019). Variations in gut bacterial communities of hooded crane (*Grus monacha*) over spatial–temporal scales. PeerJ.

[14] Eckburg P., Bik E.M., Bernstein C.N., Purdom E., Dethlefsen L., Sargent M., Gill S.R., Nelson K.E., A Relman D. (2005). Diversity of the human intestinal microbial flora. Science.

[15] Moeller A.H., Suzuki T.A., Phifer-Rixey M., Nachman M.W. (2018). Transmission modes of the mammalian gut microbiota. Science.

[16] Pai H.H., Chen W.C., Peng C.F. (2005). Isolation of bacteria with antibiotic resistance from household cockroaches (*Periplaneta americana*, and *Blattella germanica*). Acta Trop..

[17] Warnecke F., Luginbühl P., Ivanova N., Ghassemian M., Richardson T.H., Stege J.T., Cayouette M., McHardy A.C., Djordjevic G., Aboushadi N. (2007). Metagenomic and fuctional analysis of hindgut microbiota of a wood–feeding higher termite. Nature.

[18] Hamdi C., Balloi A., Essanaa J., Gonella E. (2011). Gut microbiome dysbiosis and honeybee health. J. Appl. Entomol..

[19] Koch H., Schmid-Hempel P. (2011). Socially transmitted gut microbiota protect bumble bees against an intestinal parasite. Proc. Natl. Acad. Sci. USA.

[20] Lee F.J., Rusch D.B., Stewart F.J., Mattila H.R., Newton I.L.G. (2015). Saccharide breakdown and fermentation by the honey bee gut microbiome. Environ. Microbiol..

[21] Douglas A.E. (2015). Multiorganismal insects: Diversity and function of resident microorganisms. Annu. Rev. Entomol..

[22] Scully E.D., Geib S.M., Carlson J.E., Tien M., McKenna D., Hoover K. (2014). Functional genomics and microbiome profiling of the Asian longhorned beetle (*Anoplophora glabripenni*s) reveal insights into the digestive physiology and nutritional ecology of wood feeding beetles. BMC Genom..

[23] Hernández N., Escudero J.A., Millán A.S., González-Zorn B., Lobo J.M., Verdú J.R., Suárez M. (2015). Culturable aerobic and facultative bacteria from the gut of the polyphagic dung beetle *Thorectes lusitanicus*. Insect Sci..

[24] Kaltenpoth M., Engl T. (2014). Defensive microbial symbionts in Hymenoptera. Funct. Ecol..

[25] Songvorawit N., Butcher B.A., Chaisuekul C. (2017). Decaying wood preference of stag beetles (Coleoptera: Lucanidae) in a tropical dry-evergreen forest. Environ. Entomol..

[26] Handique G., Phukan A., Bhattacharyya B., Baruah A.A., Rahman S.W., Baruah R. (2017). Characterization of cellulose degrading bacteria from the larval gut of the white grub beetle *Lepidiota mansueta* (Coleoptera: Scarabaeidae). Arch. Insect Biochem. Physiol..

[27] Tini M., Bardiani M., Chiari S., Campanaro A., Maurizi E., Toni I., Mason F., Audisio P.A., Carpaneto G.M. (2018). Use of space and dispersal ability of a flagship saproxylic insect: A telemetric study of the stag beetle (*Lucanus cervus*) in a relict lowland forest. Insect Conserv. Diver..

[28] Tanahashi M., Kubota K., Matsushita N., Togashi K. (2010). Discovery of mycangia and the associated xylose-fermenting yeasts in stag beetles (Coleoptera: Lucanidae). Naturwissenschaften.

[29] Tanahashi M., Kim J.K., Watanabe K., Fukatsu T., Kubota K. (2017). Specificity and genetic diversity of xylose-fermenting *Scheffersomyces* yeasts associated with small blue stag beetles of the genus *Platycerus* in East Asia. Mycologia.

[30] Rowland J.M., Emlen D.J. (2009). Two thresholds, three male forms result in facultative male trimorphism in beetles. Science.

[31] Matsumoto K., Knell R.J. (2017). Diverse and complex male polymorphisms in *Odontolabis* stag beetles (Coleoptera: Lucanidae). Sci. Rep..

[32] Gotoh H., Miyakawa H., Ishikawa A., Ishikawa Y., Sugime Y., Emlen D.J., Lavine L.C., Miura T. (2014). Developmental link between sex and nutrition; *doublesex* regulates sex–specific mandible growth via juvenile hormone signaling in stag beetles. PLoS Genet..

[33] Goyens J., Dirckx J., Aerts P. (2016). Jaw morphology and fighting forces in stag beetles. J. Exp. Biol..

[34] Mills M.R., Nemri R.S., Carlson E.A., Wilde W., Gotoh H., Lavine L.C., Swanson B.O. (2016). Functional mechanics of beetle mandibles: Honest signaling in a sexually selected system. J. Exp. Zool. Part A.

[35] Romiti F., DeZan L.R., Piras P., Carpaneto G.M. (2016). Shape variation of mandible and head in *Lucanus cervus* (Coleoptera: Lucanidae): A comparison of morphometric approaches. Biol. J. Linn. Soc..

[36] Jiang Y., Sun B.H., Cao Y.Y., Zhai Y.N., Wan X. (2018). Diversity of gut bacterial communities in male adults of *Odontolabis fallaciosa* (Coleoptera: Scarabaeoidea: Lucanidae) with different mandibular forms. Acta Entomol. Sin..

[37] Muyzer G., Teske A., Wirsen C.O., Jannasch H.W. (1995). Phylogenetic relationships of *Thiomicrospira* species and their identification in deep-sea hydrothermal vent samples by denaturing gradient gel electrophoresis of 16S rDNA fragments. Arch. Microbiol..

[38] Magoč T., Salzberg S.L. (2011). FLASH: Fast length adjustment of short reads to improve genome assemblies. Bioinformatics.

[39] Caporaso J.G., Kuczynski J., Stombaugh J., Bittinger K., Bushman F.D., Costello E.K., Fierer N., Peña A.G., Goodrich J.K., Gordon J.I. (2010). QIIME allows analysis of high–throughput community sequencing data. Nat. Methods.

[40] Xu L.T., Lu M., Xu D.D., Chen L., Sun J.H. (2016). Sexual variation of bacterial micr*o*biota of *Dendroctonus valens* guts and frass in relation to verbenone production. J. Insect Physiol..

[41] Briones-Roblero C.I., Rodríguez-Díaz R., Santiago-Cruz J.A., Zúñiga G., Rivera-Orduña F.N. (2016). Degradation capacities of bacteria and yeasts isolated from the gut of *Dendroctonus rhizophagus* (Curculionidae: Scolytinae). Folia Microbiol..

[42] Mason C.J., Lowe-Power T.M., Rubert-Nason K.F., Lindroth R.L., Raffa K.F. (2016). Interactions between bacteria and aspen defense chemicals at the phyllosphere–herbivore interface. J. Chem. Ecol..

[43] Han G., Lee H.J., Jeong S.E., Jeon C.O., Hyun S. (2017). Comparative Analysis of *Drosophila melanogaster* Gut Microbiota with Respect to Host Strain, Sex, and Age. Microb. Ecol..

[44] Mason C.J., Campbell A.M., Scully E.D., Hoover K. (2019). Bacterial and Fungal Midgut Community Dynamics and Transfer Between Mother and Brood in the Asian Longhorned Beetle (*Anoplophora glabripennis*), an Invasive Xylophage. Microb. Ecol..

[45] Shukla S., Vogel H., Heckel D.G., Vilcinskas A., Kaltenpoth M. (2018). Burying beetles regulate the microbiome of carcasses and use it to transmit a core microbiota to their offspring. Mol. Ecol..

[46] Tanahashi M., Ikeda H., Kubota K. (2018). Elementary budget of stag beetle larvae associated with selective utilization of nitrogen in decaying wood. Sci. Nat..

[47] Nicholson J.K., Holmes E., Kinross J., Burcelin R., Gibson G., Jia W., Pettersson S. (2012). Host–gut microbiota metabolic interactions. Science.

[48] Jacobs C.G.C., Steiger S., Heckel D.G., Wielsch N., Vilcinskas A., Vogel H. (2016). Sex, offspring and carcass determine antimicrobial peptide expression in the burying beetle. Sci. Rep..

[49] Kalpesh G.P., Nikolaos T.P. (2019). Microbiome and Metabolome in Diagnosis, Therapy, and other Strategic Applications. Chapter 28: The Microbiome and Metabolome in Alcoholic Liver Disease.

[50] Flint H.J., Bayer E.A., Rincon M.T., Lamed R., White B.A. (2008). Polysaccharide utilization by gut bacteria: Potential for new insights from genomic analysis. Nat. Rev. Microbiol..

[51] Han G.G., Kim E.B., Lee J., Lee J.Y., Jin G., Park J., Huh C.S., Kwon I.K., Kil D.Y., Choi Y.J. (2016). Relationship between the microbiota in different sections of the gastrointestinal tract, and the body weight of broiler chickens. SpringerPlus.

[52] Tap J., Mondot S., Levenze F., Pelletler E., Caron C., Furet J.P., Ugarte E., Muñoz-Tamayo R., Paslier D.L.E., Nalin R. (2009). Towards the human intestinal microbiota phylogenetic core. Environ. Microbiol..

[53] Kijimoto T., Moczek A.P. (2016). Hedgehog signaling enables nutrition–responsive inhibition of an alternative morph in a polyphenic beetle. Proc. Natl. Acad. Sci. USA.

[54] Turnbaugh P.J., Ley R.E., Mahowald M.A., Magrini V., Mardis E.R., Gordon J.I. (2006). An obesity-associated gut microbiome with increased capacity for energy harvest. Nature.

[55] Ng S.H., Stat M., Bunce M., Simmons L.W. (2018). The influence of diet and environment on the gut microbial community of field crickets. Ecol. Evol..

[56] Yu Z., Shen J., Li Z., Yao J., Li W., Xue L., Vandenberg L.N., Yin D. (2020). Obesogenic Effect of Sulfamethoxazole on *Drosophila melanogaster* with Simultaneous Disturbances on Eclosion Rhythm, Glucolipid Metabolism, and Microbiota. Environ. Sci. Technol..

