# Supplementary material for: Divergence in Gut Bacterial Community Structure between Male and Female Stag Beetles Odontolabis fallaciosa (Coleoptera, Lucanidae)
Source: Animals (Basel). 2020 Dec 9;10(12):2352. doi: 10.3390/ani10122352 (PMC7764088; doi:10.3390/ani10122352)
Supplement: Supplementary file 1 [file animals-10-02352-s001.pdf]

Supporting Information

Table S1: Indicator bacterial species in guts of *Odontolabis fallaciosa* across different treatments. Taxonomic leaves: c, class; f, family; g, genus; s, species. LM: large-sized male beetle; MM: medium-sized male beetle; SM: small-sized male beetle; FE: female beetle.

Treatment	Indicator species	Taxonomy	Indicator value	*P*	Relative abundance (%)
**LM**	OTU2498	g__*Caloramator*	0.518	0.022	3.344
	OTU1285	f__Ruminococcaceae	0.498	0.017	2.999
	OTU1916	g__*Enterococcus*	0.418	0.027	1.046
	OTU946	g__*Dysgonomonas*	0.476	0.046	0.663
	OTU1910	o__Bacteroidales	0.480	0.017	0.599
	OTU1908	g__*Ruminococcus*	0.508	0.017	0.423
	OTU1529	o__Nitrospirales	0.483	0.018	0.369
	OTU1955	f__Lachnospiraceae	0.615	0.002	0.320
	OTU2457	g__*Caloramator*	0.483	0.025	0.288
	OTU1374	f__Veillonellaceae	0.439	0.040	0.115
**MM**	OTU1190	f__Pseudomonadaceae	0.566	0.004	0.160
**SM**	OTU2925	f__Neisseriaceae	0.417	0.022	4.088
	OTU1661	g__*Dysgonomonas*	0.456	0.035	1.556
	OTU1220	g__*Marinitoga*	0.470	0.049	0.223
**FE**	OTU3378	g__*Pseudomonas*	0.809	0.015	5.006
	OTU2845	f__Enterobacteriaceae	0.416	0.014	4.228

OTU1435	c__Chthonomonadetes	0.888	0.001	3.131
OTU1299	s__*T. siberiense*	0.517	0.004	2.605
OTU1376	g__*Arcobacter*	0.666	0.001	1.474
OTU2892	f__Aeromonadaceae	0.792	0.001	0.643
OTU2937	g__*Klebsiella*	0.630	0.004	0.637
OTU1874	g__*Desulfovibrio*	0.781	0.002	0.604
OTU1907	g__*Fusobacterium*	0.600	0.002	0.601
OTU1865	c__Alphaproteobacteria	0.583	0.006	0.449
OTU1159	f__Rhizobiaceae	0.693	0.002	0.394
OTU1249	g__*Coprothermobacter*	0.637	0.021	0.350
OTU3053	f__Phormidiaceae	0.597	0.021	0.331
OTU1170	f__Erythrobacteraceae	0.658	0.003	0.317
OTU1168	f__Alteromonadaceae	0.467	0.002	0.312
OTU1183	g__*Chryseobacterium*	0.570	0.038	0.303

Table S2: The multivariate variance across the different treatments was evaluated by betadisper analysis. Differences in gut bacterial community composition across the different treatments examined by the dissimilarity test of ANOSIM. LM: large-sized male beetle; MM: medium-sized male beetle; SM: small-sized male beetle; FE: female beetle.

**Treatment**	**Betadisper**		**ANOSIM**	
	F	*P*	r	*P*
LM *vs* MM	3.831	0.078	0.197	0.101
LM *vs* SM	0.122	0.731	0.040	0.329
LM *vs* FE	1.838	0.186	**0.420**	**0.001**
MM *vs* SM	3.232	0.094	0.155	0.942
MM *vs* FE	4.197	0.063	**0.234**	**0.011**
SM *vs* FE	0.513	0.482	**0.267**	**0.009**

Figure S1: The mandible shape and body size of *O. fallaciosa* between two sexes, and among three male morphs. LM: large-sized male beetle; MM: medium-

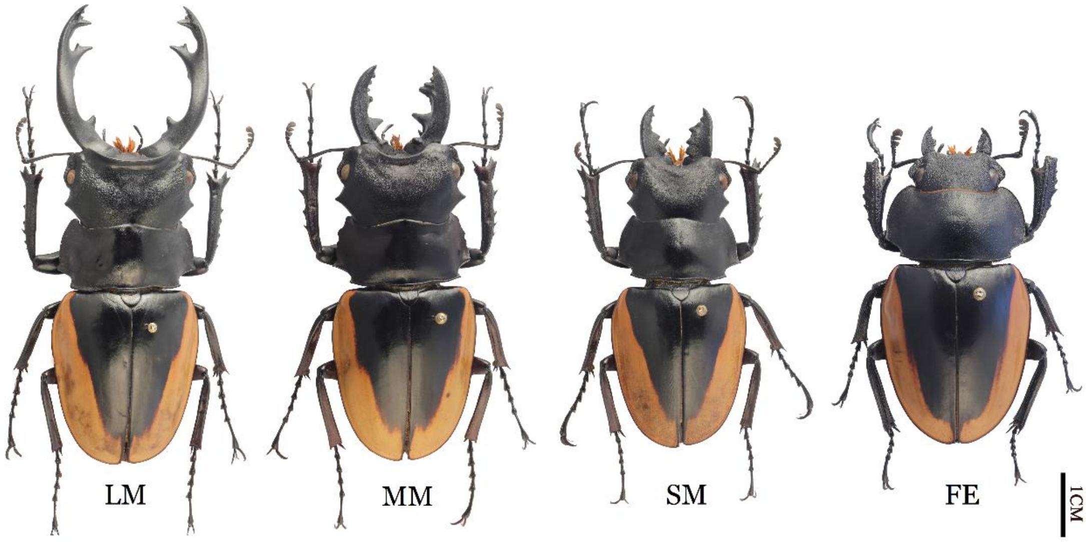

sized male beetle; SM: small-sized male beetle; FE: female beetle.

Figure S2: Venn diagram showing the co-occurence of the OTUs among samples from different treatments. Numbers inside the Venn diagram indicate unique and shared OTUs. OTU, operational taxonomic unit. LM: large-sized

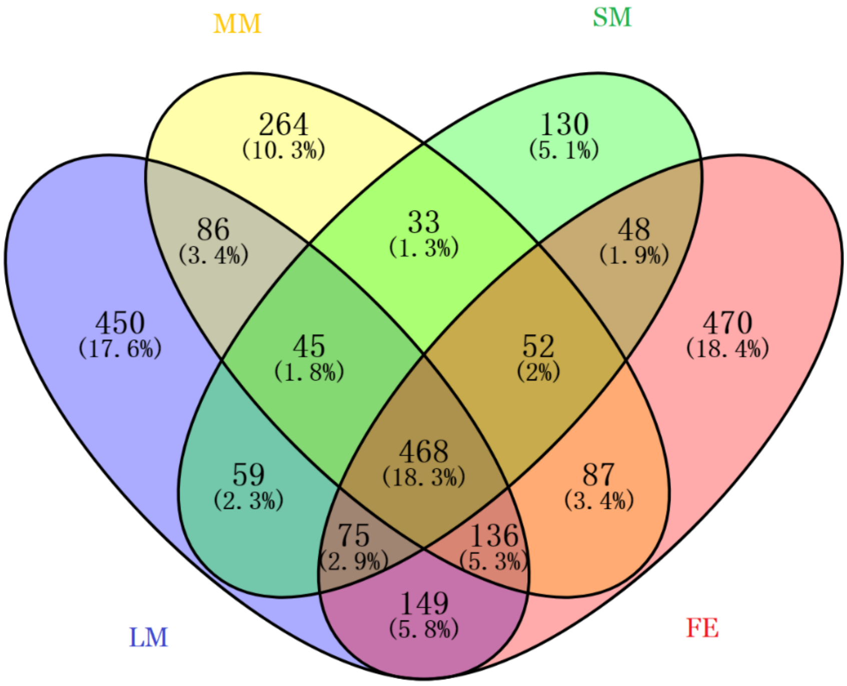

male beetle; MM: medium-sized male beetle; SM: small-sized male beetle; FE: female beetle.
